# Diagnostic delay and associated factors among patients with pulmonary tuberculosis in Dar es Salaam, Tanzania

**DOI:** 10.1186/s40249-017-0276-4

**Published:** 2017-03-24

**Authors:** Khadija Said, Jerry Hella, Grace Mhalu, Mary Chiryankubi, Edward Masika, Thomas Maroa, Francis Mhimbira, Neema Kapalata, Lukas Fenner

**Affiliations:** 1 Ifakara Health Institute, Bagamoyo Research and Training Centre (BRTC), P O Box 74, Bagamoyo, Tanzania; 2 Swiss Tropical and Public Health Institute, Socinstrasse 57, 4002 Basel, Switzerland; 3 University of Basel, Petersplatz 1, 4003 Basel, Switzerland; 4 National Tuberculosis and Leprosy Programme, Dar es Salaam, Tanzania; 5 Institute of Social and Preventive Medicine, University of Bern, Bern, Switzerland

**Keywords:** Tanzania, Tuberculosis, Diagnostic delay, Health-seeking, Geographic information system, Pharmacy, Transmission

## Abstract

**Background:**

Tanzania is among the 30 countries with the highest tuberculosis (TB) burdens. Because TB has a long infectious period, early diagnosis is not only important for reducing transmission, but also for improving treatment outcomes. We assessed diagnostic delay and associated factors among infectious TB patients.

**Methods:**

We interviewed new smear-positive adult pulmonary TB patients enrolled in an ongoing TB cohort study in Dar es Salaam, Tanzania, between November 2013 and June 2015. TB patients were interviewed to collect information on socio-demographics, socio-economic status, health-seeking behaviour, and residential geocodes. We categorized diagnostic delay into ≤ 3 or > 3 weeks. We used logistic regression models to identify risk factors for diagnostic delay, presented as crude (*OR*) and adjusted Odds Ratios (a*OR*). We also assessed association between geographical distance (incremental increase of 500 meters between household and the nearest pharmacy) with binary outcomes.

**Results:**

We analysed 513 patients with a median age of 34 years (interquartile range 27–41); 353 (69%) were men. Overall, 444 (87%) reported seeking care from health care providers prior to TB diagnosis, of whom 211 (48%) sought care > 2 times. Only six (1%) visited traditional healers before TB diagnosis. Diagnostic delay was positively associated with absence of chest pain (a*OR* = 7.97, 95% confidence intervals [CI]: 3.15–20.19; *P* < 0.001), and presence of hemoptysis (a*OR* = 25.37, 95% *CI*: 11.15–57.74; *P* < 0.001) and negatively associated with use of medication prior to TB diagnosis (a*OR* = 0.31, 95% *CI*: 0.14–0.71; *P* = 0.01). Age, sex, HIV status, education level, household income, and visiting health care facilities (HCFs) were not associated with diagnostic delay. Patients living far from pharmacies were less likely to visit a HCF (incremental increase of distance versus visit to any facility: *OR* = 0.51, 95% *CI*: 0.28–0.96; *P* = 0.037).

**Conclusions:**

TB diagnostic delay was common in Dar es Salaam, and was more likely among patients without prior use of medication and presenting with hemoptysis. Geographical distance to HCFs may have an impact on health-seeking behaviour. Increasing community awareness of TB signs and symptoms could further reduce diagnostic delays and interrupt TB transmission.

**Electronic supplementary material:**

The online version of this article (doi:10.1186/s40249-017-0276-4) contains supplementary material, which is available to authorized users.

## Multilingual abstracts

Please see Additional file 1 for translations of the abstract into the six official working languages of the United Nations.

## Background

Tuberculosis (TB) control remains a major public health challenge in low-income countries with a high incidence of TB, particularly where HIV prevalence is also high [1]. In these settings, TB accounts for approximately 40% of adult deaths. In almost half of these cases, the disease remains undiagnosed until death [2]. TB patients are often diagnosed at the later stages of the disease, due to health-seeking behaviour, inappropriate diagnostic investigations requested by health care providers, and limited diagnostic capacities at health care facilities (HCFs) [3, 4]. Patients experiencing TB symptoms may initially seek relief by using self-prescribed medication or by consulting a health care provider who does not request TB investigations despite repeated visits [5]. The economic burden of seeking care remains a barrier for TB patients [6].

Delaying diagnosis and treatment of TB has important consequences for disease control at both the individual as well as the community level. At the individual level, a patient with a delayed diagnosis of TB risks advanced disease states and worse treatment outcomes. At community level, a patient with delayed diagnosis is infectious to close contacts; an untreated smear-positive TB case infects approximately 15 people annually [7, 8]. Delay in seeking care, therefore, promotes continued TB transmission [9]. The complex pathway to care, from the onset of symptoms to diagnosis and treatment, may result in delays in seeking care and contribute to patient morbidity and mortality [4, 10].

Tanzania is among the 30 countries with the highest TB burdens [11]. TB case detection in Tanzania largely relies on passive case detection when patients present themselves to HCFs, which likely leads to longer diagnostic delays [12]. The first national TB prevalence survey in Tanzania in 2012 showed that only 30% of people with TB symptoms sought health care and almost half of these did not undergo diagnostic procedures for TB [4]. To better understand this situation, we studied the extent of diagnostic delay of TB, its associated risk factors and factors contributing to health-seeking behaviour among newly diagnosed smear-positive adult pulmonary TB (PTB) patients in Dar es Salaam, an urban region in Tanzania with a high TB burden.

## Methods

### Study setting and study population

We collected data from an ongoing prospective cohort of smear-positive adult PTB patients (≥18 years) in Dar es Salaam, Tanzania (TB-DAR). TB-DAR was initiated in November 2013 to study the epidemiology and molecular epidemiology of TB in the densely populated Temeke district in Tanzania’s commercial capital, Dar es Salaam. Dar es Salaam, with a population of about 4.4 million and rapid urbanization [13], accounted for nearly 22% of the 65 732 TB cases notified in 2013 to the National Tuberculosis and Leprosy Programme (NTLP) in Tanzania [14, 15]. The Temeke district has approximately 1.4 million people and reported 4,373 TB cases in 2014 [15]. The main sources of income in the district are food crop sales and small businesses [16].

Eligibility criteria for TB-DAR study participants were as follows: 1) ≥18 years of age at recruitment, 2) sputum microscopy smear-positive TB (quantification grading at least scanty), 3) residency in Wailes I and II sub-districts of Temeke and 4) attends the TB clinic at the Temeke District Hospital or one of its associated TB satellite treatment centers, Tambukareli and Pasada. Patients with confirmed TB were started on standard TB treatment regimen within 1 day of diagnosis, as per the national guidelines [17]. Diagnostic and treatment services for TB are provided free of charge by the NTLP.

**Fig. 1:**
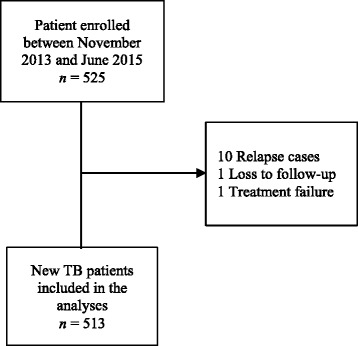
Flow chart of patient selection

For this analysis, we included newly diagnosed smear-positive adult PTB cases who were enrolled between November 2013 and June 2015. The selection of eligible patients is shown in Fig. 1. Of the 525 smear-positive PTB patients enrolled in the TB-DAR Study during this time period, we excluded 12 (2.3%) patients for the following reasons: 10 (2%) relapse patients, 1 (0.2%) loss to follow-up and 1 (0.2%) treatment failure (Fig. 1).

According to the national guidelines [17], presumptive PTB cases (outpatient or inpatient) are counseled to bring sputum samples to the laboratory; patients with a smear-positive result or smear-negative TB cases with clinical features suggestive of TB (and/or based on additional tests such as chest X-ray) are immediately started on TB treatment.

### Data collection and definitions

Clinical officers with extensive experience in clinical research interviewed patients using standardized questionnaires [18]. Officers collected data on socio-demographic and socio-economic characteristics and on health-seeking behaviour at the time of enrolment. In ascertaining signs and symptoms suggestive of TB, we posed specific questions (patient ever having felt sick of any symptom such as coughing, chest pain, fever, weight loss, hemoptysis, night sweats) and patients were asked to recall the duration of symptoms (in weeks) [19]. Duration of diagnostic delay was calculated based on the longest reported TB-related symptom.

A new adult TB case was defined as a patient aged ≥ 18 years with newly diagnosed smear-positive (at least scanty) pulmonary TB. Sputum microscopy was performed using fluorescent light-emitting diode (LED) microscopy and the quantitative scoring system was based on the number of acid-fast bacilli (AFB) according to the national guidelines [17, 20]: scanty, 1+, 2+, and 3+. Diagnostic delay was defined as the time from onset of any TB-related symptom (patient ever having felt sick of any symptom such as coughing, chest pain, fever, weight loss, hemoptysis and night sweat) until the time of TB diagnosis [3, 19, 21, 22]. Health Care Facilities (HCFs) were defined as places that provide health care, including hospitals, health centers and dispensaries. Prior medication was defined as the use of any self-prescribed or prescribed medication prior to TB diagnosis and any medication other than anti-TB, antiretroviral therapy (ART) or co-trimoxazole (for patients co-infected with HIV). Multiple HCF visits were defined as seeking health care from formal health providers more than twice.

### Statistical analysis

Descriptive analyses were performed to summarize the data. For the purpose of this study, we categorized diagnostic delay into two categories: 3 weeks or less and more than 3 weeks. We considered 3 weeks as a cutoff for diagnostic delay based on the distribution of the delay in our study population (Additional file 2: Figure S1) and the range of diagnostic delay reported in a systematic review [23]. Associations between dependent variables (delay of ≤ 3 and > 3 weeks) and independent variables (such as age, sex, occupation, household income, household size, TB symptoms, prior use of medication, and number of HCF visits) were analyzed using logistic regression models. Variables were included in the multivariate model if thought to be clinically or socially relevant or had a *P*-value of < 0.05 following univariate analysis. Result are presented as crude (*OR*) and adjusted odds ratios (a*OR*). All analyses were performed in STATA software version 13.1 (Stata Corporation, Collage Station, Texas, USA).

### Geographical analysis

We collected geocodes using Android tablets (Samsung) for all known pharmacies, dispensaries (public/private), and hospitals, identified during extensive fieldwork in the study area. In addition, all study participants’ households were geocoded. We generated maps using the open source software QGIS version 2.10.1 [24]. We then calculated the Euclidean distances in meters as a linear distance matrix between participants’ households and the nearest pharmacy. The resulting Euclidean distances were then imported into STATA software for further analyses. To overcome separation in conventional logistic models, we created penalized likelihood models using the firth logit command in STATA and assessed the association between an incremental increase of 500 meters (distance between household and the nearest pharmacy) with binary outcomes (prior use of medication, diagnostic delay and any visit to a HCF) [25]. Results were presented as unadjusted *OR*s (Fig. 3).

## Results

### Patient characteristics

We analysed 513 newly diagnosed smear-positive adult PTB patients. The median age was 34 years (interquartile range [IQR]: 27–41 years, range 18–79); 353 (69%) were men and 147 (29%) were HIV-positive (Table 1). The proportion of HIV was higher among women compared to men (43%, versus 22%; *P* < 0.001). Of the 147 HIV-positive patients, 37 (25%) were on ART prior to TB diagnosis and 86 (59%) started ART within 7 weeks of TB diagnosis; ART information was unknown for 24 (16%) patients. Overall, 92 patients (18%) had no formal education. More than half of the patients (341; 66%) were semi-skilled workers. Most patients lived in rented houses (288; 56%). The median household size was three persons (IQR: 2–4 persons) and 408 (80%) patients earned < 200 USD per month.

**Table 1:** Patient characteristics of new smear-positive adult pulmonary tuberculosis (TB) cases in the Temeke District, Dar es Salaam, Tanzania

Variable	All	Delay duration		Prior medication		HC facility visits	
	*n* = 513	(weeks)					
*n* (%)		≤ 3	> 3	Not used	Used	≤ 2 visits	> 2 visits
Total	513 (100)	406 (79)	107 (21)	52 (10)	461 (90)	297 (58)	216 (42)
Age, years							
18–24	91 (18)	73 (18)	18 (17)	6 (12)	85 (18)	58 (20)	33 (15)
25–45	333 (65)	264 (65)	69 (64)	37 (71)	296 (64)	199 (67)	134 (59)
> 45	89 (17)	69 (17)	20 (19)	9 (17)	80 (17)	40 (13)	49 (23)
Sex							
Male	353 (69)	280 (69)	73 (68)	38 (73)	315 (68)	209 (70)	144 (67)
Female	160 (31)	126 (31)	34 (32)	14 (27)	146 (32)	88 (30)	72 (33)
HIV status							
Positive	146 (28)	119 (29)	27 (25)	16 (31)	130 (28)	73 (25)	73 (34)
Negative	367 (72)	287 (71)	80 (75)	36 (69)	331 (72)	224 (75)	143 (66)
Education							
No education	92 (18)	73 (18)	19 (18)	8 (15)	84 (18)	46 (15)	46 (21)
Primary/Secondary	404 (79)	316 (78)	88 (82)	44 (85)	360 (78)	243 (82)	161 (75)
University	17 (3)	17 (4)	0 (0)	0 (0)	17 (4)	8 (3)	9 (4)
Occupation							
Unemployed or h/wife	103 (20)	86 (21)	17 (16)	4 (8)	99 (21)	46 (15)	57 (26)
Unskilled labour	69 (13)	52 (13)	17 (16)	11 (21)	58 (13)	38 (13)	31 (14)
Semiskilled labour	341 (67)	268 (66)	73 (68)	37 (71)	304 (66)	213 (72)	128 (60)
Household income							
< $200 per month	408 (80)	329 (81)	79 (74)	37 (71)	371 (80)	225 (76)	183 (85)
≥ $200 per month	105 (20)	77 (19)	28 (26)	15 (29)	90 (20)	72 (24)	33 (15)
BMI category, kg/m^2^							
< 18.5	36 (7)	29 (7)	7 (7)	3 (6)	33 (7)	25 (8)	11 (5)
18–24.9	273 (53)	204 (50)	69 (64)	20 (38)	253 (55)	163 (55)	110 (51)
25–29.9	154 (30)	129 (32)	25 (23)	19 (37)	135 (29)	78 (26)	76 (35)
> 30	50 (210)	44 (11)	6 (6)	10 (19)	40 (9)	31 (11)	19 (9)
Household size, persons							
≥ 4	137 (27)	123 (30)	14 (13)	3 (6)	134 (29)	56 (19)	81 (37)
2–3	331 (64)	256 (63)	75 (70)	43 (83)	288 (63)	208 (70)	123 (57)
Single	45 (9)	27 (7)	18 (17)	6 (12)	39 (8)	33 (11)	12 (6)
House ownership							
Own	225 (44)	178 (79)	47 (21)	17 (33)	208 (45)	127 (43)	98 (45)
Rented	288 (56)	228 (79)	60 (21)	35 (67)	253 (55)	170 (57)	118 (55)
Coughing							
No	2 (0.5)	2 (0.5)	0	0 (0)	2 (0.4)	1 (0.3)	1 (0.5)
Yes	511 (99.5)	404 (99.5)	107 (100)	52 (100)	459 (99.6)	296 (99.7)	215 (99.5)
Fever							
No	38 (7)	37 (9)	1 (1)	3 (6)	35 (8)	33 (11)	5 (2)
Yes	475 (93)	369 (91)	106 (99)	49 (94)	426 (92)	264 (89)	211 (98)
Chest pain							
No	102 (20)	69 (17)	33 (31)	40 (77)	62 (14)	94 (32)	8 (4)
Yes	411 (80)	337 (83)	74 (69)	12 (23)	399 (87)	203 (68)	208 (96)
Haemoptysis							
No	336 (71)	324 (80)	42 (39)	52 (100)	314 (68)	185 (62)	181 (84)
Yes	147 (29)	82 (20)	65 (61)	0	147 (32)	112 (38)	35 (16)
Night sweat							
No	26 (5)	25 (6)	1 (1)	1 (2)	25 (5)	24 (8)	2 (1)
Yes	487 (95)	381 (94)	106 (99)	51 (98)	436 (95)	273 (92)	214 (99)
Unexplained weight loss							
No	20 (4)	18 (4)	2 (2)	1 (2)	19 (4)	15 (5)	5 (2)
Yes	493 (96)	388 (96)	105 (98)	51 (98)	442 (96)	282 (95)	211 (98)

*BMI* body mass index, *h/wife* housewife, *TB* tuberculosis, *HC facility* health care facility

The most commonly reported symptom was coughing (511 patients; 99.6%), followed by weight loss (493; 96%), night sweats (487; 94%), fever (475; 93%), chest pain (411; 80%), and hemoptysis (147; 29%) (Table 1). The median duration of cough was 3 weeks (IQR: 3.0–3.0). All other symptoms had a median duration of 2 weeks (IQR: 1.0–3.0).

### Health-seeking behaviour and diagnostic delay

About 444 (87%) patients reported seeking care from at least one of the formal health care providers; of these, 211 (48%) sought care at a HCF three times or more in relation to TB symptoms prior to TB diagnosis. Only six (1%) reported to have sought care from traditional healers and 63 (12%) did not seek care until they presented to the HCFs of the study recruitment centers, where they were diagnosed with TB. The median age of those who sought care three times or more was 35 years (IQR: 29–44). More men than women sought formal care three times or more (67% versus 33%). Almost all patients reported cough (Table 1). The majority of patients reported having taken medication prior to TB diagnosis (461 patients; 90%); amoxicillin was the most common drug of choice (432; 94%), followed by ciprofloxacin (5; 1%).

The median diagnostic delay time was 3 weeks (IQR: 3.0–3.0, range 1–45 weeks); 28 (5%) patients had a diagnostic delay of more than 4 weeks. By the third week of presenting with TB symptoms, 442 (86%) patients had been diagnosed with the disease. Additional file 2: Figure S1 shows the distribution of the diagnostic delay in the study population.

### Patient factors associated with diagnostic delay

In the multivariate analysis, diagnostic delay of > 3 weeks was more likely among patients who did not have chest pain (a*OR* = 7.97, 95% *CI*: 3.15–20.19; *P* < 0.001), and who presented with hemoptysis (a*OR* = 25.37, 95% *CI*: 11.15–57.74; *P* < 0.001), and less likely among patients who used medication prior to TB diagnosis (a*OR* = 0.31, 95% *CI*: 0.14–0.71; *P* = 0.03) as shown in Table 2. Age, sex, HIV status, occupation, level of income, household size, and visits to HCF were not associated with diagnostic delay (Table 2).

**Table 2:** Associations of diagnosis delay (defined as > 3 weeks) with socio-demographic and clinical characteristics among new pulmonary TB patients

Characteristic	Cases	Unadjusted		Adjusted	
	*n* (%)	*OR* (95% *CI*)	*P*-value	a*OR* (95% *CI*)	*P*-value
Age, years			0.7		0.7
18–24	91 (18)	1		1	
25–45	333 (65)	1.06 (0.59–1.89)		0.81 (0.39–1.67)	
> 45	189 (17)	1.18 (0.57–2.41)		1.08 (0.44–2.66)	
Sex			0.9		0.6
Female	160 (31)	1		1	
Male	353 (69)	0.97 (0.61–1.53)		0.84 (0.46–1.51)	
HIV status			0.4		0.5
Negative	366 (71)	1		1	
Positive	147 (29)	0.81 (0.50–1.30)		0.83 (0.46–1.52)	
Occupation			0.4		0.1
Unemployed, or h/wife	103 (20)	1		1	
Unskilled labor	69 (13)	1.65 (0.78–3.52)		1.24 (0.48–3.26)	
Semiskilled labor	341 (67)	1.38 (0.77–2.46)		0.69 (0.32–1.51)	
Household income			0.1		0.3
< $200 per month	408 (80)	1		1	
≥ $200 per month	105 (20)	1.51 (0.92–2.49)		1.34 (0.73–2.47)	
Household size, persons			< 0.001		0.2
≥ 4	137 (27)	1		1	
2–3	331 (64)	2.57 (1.40–4.74)		1.38 (0.67–2.83)	
Single	45 (9)	5.86 (2.60–13.21)		1.81 (0.71–4.59)	
Visits to a HCF			< 0.001		0.9
> 2	216 (42)	1		1	
≤ 2	297 (58)	3.31 (2.01–5.46)		0.99 (0.52–1.90)	
Prior purchase of medication			0.001		0.01
No	52 (10)	1		1	
Yes	461 (90)	0.37 (0.20–0.68)		0.31 (0.14–0.71)	
Chest pain			0.002		<0.001
Yes	411 (80)	1		1	
No	102 (20)	2.18 (1.34–3.54)		7.97 (3.15–20.19)	
Hemoptysis			<0.001		<0.001
No	366 (71)	1		1	
Yes	147 (29)	6.11 (3.87–9.66)		25.37 (11.15–57.74)	

*OR* odds ratio, a*OR* adjusted odds ratio, 95% *CI*, 95% confidence interval, *h/wife* housewife, *HCF* health care facility

Model was adjusted for age, sex, HIV status, occupation, house hold income, household size, visit to HCF, prior use of medication, chest pain and hemoptysis

### Health-seeking behaviour and geographical distance to pharmacies

**Fig. 2:**
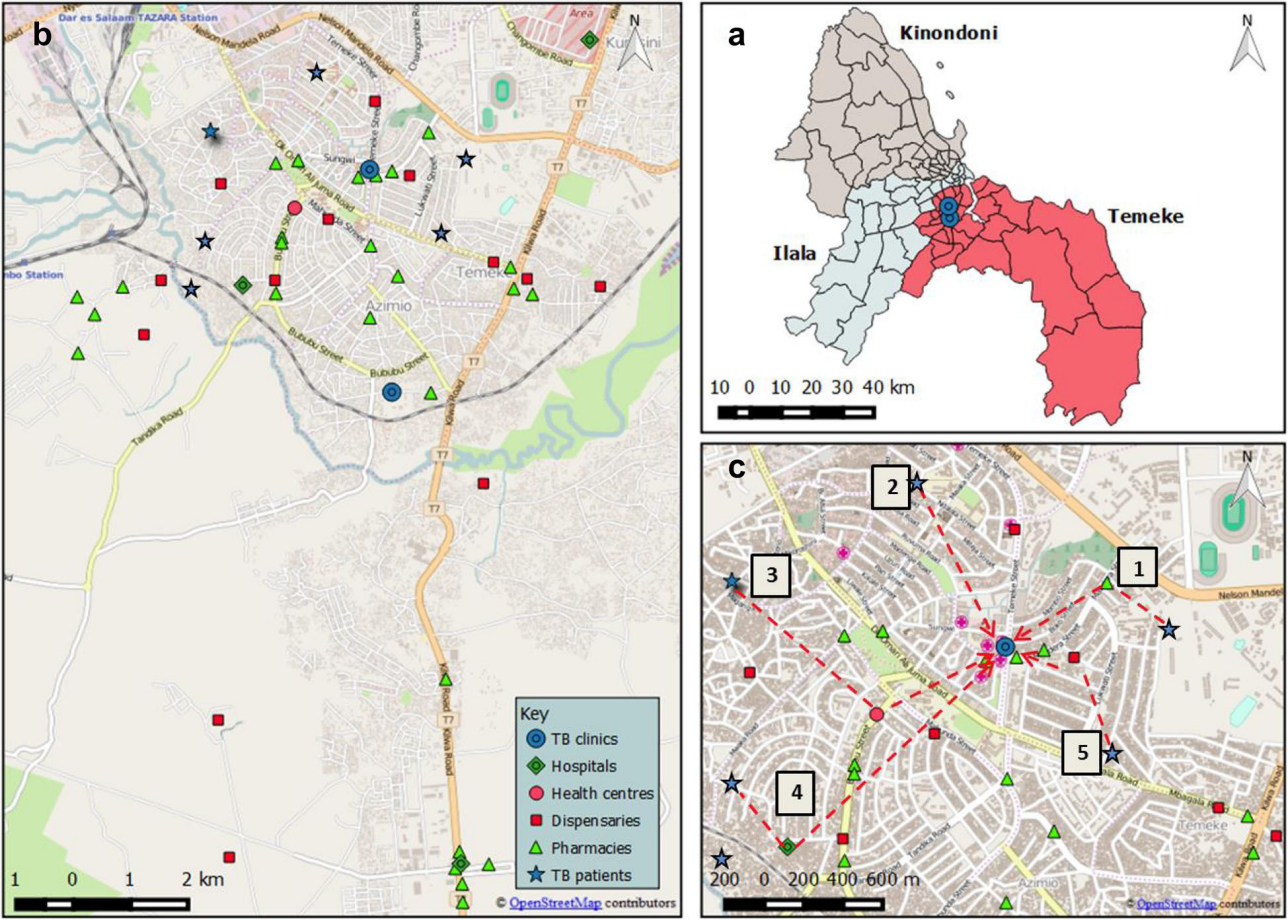
Geographical analyses of health care facilities (HCFs) and pathways to care of patients with tuberculosis (TB) symptoms in the study area, Temeke District, Dar es Salaam, Tanzania. Panel **a**: Localization of the two governmental TB clinics which serve as recruitment sites in the study area (in *red*). Panel **b**: Spatial distribution of pharmacies and HCFs in the study area. Panel **c**: Five examples of possible pathways to care of patients with TB symptoms seeking care. Various types of HCFs as the entry point into the health care system (single or multiple visits) until final diagnosis at the TB clinic are presented

**Fig. 3:**
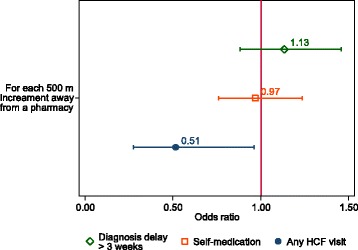
Association between health-seeking behaviour and geographical distances to pharmacies. The regression coefficient plot (with corresponding 95% confidence intervals) shows the association of the Euclidean distance between participants’ households to the nearest pharmacy with purchase of medication prior to TB diagnosis, diagnostic delay (> 3 weeks), and visit of any HCF before TB diagnosis. Odds Ratios (*OR*) above 1 indicate that the factor is more likely with increasing distance away from the nearest pharmacy, and an *OR* below 1 that the factor is more likely the closer the household is to a pharmacy. HCF, health care facility

The map in Fig. 2 shows the spatial distribution of pharmacies and HCFs in the study area, and offers examples of the complex pathways to care until final TB diagnosis. We counted a total of 35 pharmacies within the catchment area of the study. The median Euclidean distance from a participant’s household to the nearest pharmacy was 412.3 meters (IQR: 254–781, range 6–4 820). More than half of participants’ households (303; 59%) were found within 500 meters of their nearest pharmacy; 137 (26.7%) were between 500 and 1 000 meters to the nearest pharmacy and only 73 (14.2%) participants’ households were more than 1 000 meters away from a pharmacy. For each incremental increase of 500 meters away from a pharmacy, the odds of visiting a HCF decreased (*OR* = 0.51, 95% *CI*: 0.28–0.96; *P* = 0.037, comparing patients with any visit to a HCF versus no visit prior to TB diagnosis, Fig. 3). Geographical distance did not seem to have an effect on the use of medication prior to TB diagnosis or on the degree of diagnostic delay.

## Discussion

We studied diagnostic delay among adult infectious TB patients in an urban district of Dar es Salaam, Tanzania, with a high TB notification rate. We found that multiple visits to HCFs and prior use of prescribed or self-prescribed medication was frequent (41% and 90%) and that multiple visits were more likely among patients living closer to HCFs.

We showed that 21% of patients delayed seeking care for TB symptoms. The median diagnostic delay of 3 weeks in our study was shorter compared to other delay studies conducted in sub-Saharan Africa, Asia as well as Eastern Europe [5, 26–30]. A systematic review from low- and high-income settings reported a median total delay of 3–26 weeks [23]. Our diagnostic delay (time from onset of symptoms until diagnosis) was also shorter than the treatment delay (time from onset of symptoms until treatment) reported in a study conducted in six rural and urban districts in Tanzania (median treatment delay time was 10–14.5 weeks) [31]. In our study, we disregarded the treatment delay because all patients were strictly put on TB treatment within 1 day of confirming TB diagnosis, as per the guidelines of the NTLP.

We found that more than three quarters of patients sought formal care prior to being diagnosed with TB, with nearly half of them seeking care more than twice. This indicates that a high proportion of TB patients is seen by several different health care providers before receiving a TB diagnosis, suggesting a low suspicion index among health care providers. The first national TB prevalence survey in Tanzania reported that one third of patients suspected of having TB sought care before the survey, which confirms that many infectious TB cases are missed by the health care system in Tanzania [4]. Furthermore, a study from Uganda showed that the proportion of patients who sought care before being diagnosed with TB can be as high as 80% [32].

We also found that use of medication prior to TB diagnosis was associated with shorter diagnostic delays. This could be explained by the fact that patients who did not use medication prior to TB diagnosis could not afford any health care service, feared the stigma of being known to be sick, or did not have adequate knowledge about signs and symptoms of TB. Indeed, inadequate knowledge about TB and stigma has previously been associated with longer diagnostic delays [3, 23, 33–35]. In contrast, studies from Georgia, Ethiopia and Angola reported that the use of antibiotics prior to TB diagnosis was associated with a prolonged delay [5, 36, 37]. This difference can be explained by the fact that we defined use of medication as any medication prior to TB diagnosis, rather than antibiotics alone, as patients may not accurately remember the type of medication used in the past. Furthermore, patients may tend to wait for improvement of symptoms before seeking health care in these specific study settings and countries, but not in our setting of Dar es Salaam.

Patient factors associated with diagnostic delay may vary considerably across settings and countries [8, 28, 29, 33, 38–40]. In our study, diagnostic delay was not reduced among HIV-positive patients despite the widely implemented TB/HIV collaborative activities by the National AIDS Control Programme and the NTLP in Tanzania [17]. We also found that chest pain was associated with a shorter diagnostic delay, in contrast to studies from Afghanistan and Brazil [41, 42]. Hemoptysis, which usually presents at the later stage of disease [43], was associated with a prolonged delay in our study. Patients presenting with hemoptysis may tend to ignore other TB symptoms until the disease is advanced and hemoptysis has developed. Systematic reviews reported the opposite trend, possibly because hemoptysis may necessitate health care providers to investigate for TB early on [8, 44]. Consistent with our results, previous studies found no association of diagnostic delay with patients’ sex [33] or low-income class [8].

We also investigated the impact of the geographical distance on the health-seeking behaviour of patients with TB symptoms using geographic information system (GIS) tools. GIS is increasingly being used to describe the epidemiology of diseases and to map HCFs for strategic planning of public health measures [45–47]. We found that patients living closer to a pharmacy tended to visit facilities more frequently. This suggests that the availability of health care services (public/private) affects use by the resident population. In line with our results, a study from Tanzania showed that residing at a distance of more than 5 km from a TB diagnostic center was associated with diagnostic delay [29]. In addition, a systematic review found that a longer walking distance to a HCF was correlated with patient delay, defined as the time from onset of the first TB symptoms (usually described as the onset of persistent coughing) until the date of the first HCF consultation [44].

Our study has several limitations. First, we had to rely on patients’ recall of previous interactions with the health care system. This could have introduced a recall bias influencing the accuracy of the data. However, our interviewers were well trained in patient interviews and experienced in conducting scientific studies. Therefore, the collected information was likely to be accurate. Second, our findings may be specific to urban settings in Tanzania such as Dar es Salaam as well as to the study period and, therefore, may not be generalizable. Finally, we did not collect detailed information on the pathway to care at the individual level, such as dates and types of HCF visited, patients’ reasons for delay or socio-cultural factors underlying the delay in seeking care.

## Conclusions

In conclusion, we found that TB diagnostic delay was common in urban Tanzania, possibly due to passive case detection practice. We also found that the use of medication prior to TB diagnosis was common, which potentially poses challenges in the rational use of medication. Since many patients repeatedly accessed health care services before being diagnosed with TB, our study re-emphasizes the need for improved diagnostic capacities, as well as training and re-training of health care workers to increase awareness and timely diagnosis of TB. Consequent systematic screening for TB at HCFs at any care level could further reduce the diagnostic delay to interrupt TB transmission in the community. To support the implementation of active case detection interventions, future research should focus on health-seeking behaviour and how it is affected by cost factors and by the role of socio-cultural and other community determinants [12, 48–50].

## Additional files

Additional file 1: Multilingual abstracts in the five official working languages of the United Nations. (PDF 776 kb)

Additional file 2: Figure S1. Histogram of delay time reported among 507 TB patients. (DOCX 79 kb)

## Abbreviations

ART: Antiretroviral therapy
GIS: Geographic information system
HCF: Health care facility
HIV: Human immunodeficiency virus
IHI: Ifakara health institute
IRB: Institutional review board
NIMR: National institute of medical research
PTB: Pulmonary tuberculosis
TB: Tuberculosis

## References

[1] Cummings KJ (2007). Tuberculosis control: challenges of an ancient and ongoing epidemic. Public Health Rep.

[2] Gupta RK (2015). Prevalence of tuberculosis in post-mortem studies of HIV-infected adults and children in resource-limited settings: a systematic review and meta-analysis. Aids.

[3] World Health Organization, Diagnostic and treatment delay in tuberculosis. Geneva: WHO; 2006.

[4] Senkoro M (2015). Health care-seeking behaviour among people with cough in Tanzania: findings from a tuberculosis prevalence survey. Int J Tuberc Lung Dis.

[5] Rabin AS (2013). Prescribed and self-medication use increase delays in diagnosis of tuberculosis in the country of Georgia. Int J Tuberc Lung Dis.

[6] Chen S (2015). Are free anti-tuberculosis drugs enough? An empirical study from three cities in China. Infect Dis Poverty.

[7] World Health Organization, WHO fact sheet on tuberculosis (TB) 2015. Geneva: WHO; 2015.

[8] Storla DG, Yimer S, Bjune GA (2008). A systematic review of delay in the diagnosis and treatment of tuberculosis. BMC Public Health.

[9] Creswell J (2015). Introducing new tuberculosis diagnostics: the impact of Xpert® MTB/RIF testing on case notifications in Nepal. Int J Tuberc Lung Dis.

[10] Kapoor SK (2012). How did the TB patients reach DOTS services in Delhi? A study of patient treatment seeking behavior. PLoS ONE.

[11] World Health Organization, WHO | Global tuberculosis report 2015. Geneva: WHO; 2015.

[12] Ngadaya ES (2009). Delay in tuberculosis case detection in Pwani region, Tanzania. A cross sectional study. BMC Health Serv Res.

[13] The United Republic of Tanzania (2012). Tanzania Population and Housing Census 2012.

[14] World Health Organization, WHO | Global tuberculosis report 2014. Geneva: WHO; 2014.

[15] National Tuberculosis and Leprosy Control Programme (2014). National Tuberculosis and Leprosy Annual Report.

[16] National Bureau of Statistics (2014). Office of Regional Commissioner, Dar es Salaam Region Socio-economic Profile.

[17] Ministry of Health and Social Welfare (2013). Manual for the Management of Tuberculosis and Leprosy.

[18] Steiner A, Hella J, Grüninger S, Mhalu G, Mhimbira F, Cercamondi CI, Doulla B, Maire N, Fenner L (2016). Managing research and surveillance projects in real-time with a novel open-source eManagement tool designed for under-resourced countries. J Am Med Inform Assoc.

[19] Aoki M, Mori T, Shimao T (1985). Studies on factors influencing patient’s, doctor’s and total delay of tuberculosis case-detection in Japan. Bull Int Union Tuberc Lung Dis.

[20] Richard L, Armand Van D, Ivan B, Mark F-G (2013). The Handbook - Laboratory Diagnosis of Tuberculosis by Sputum Microscopy.

[21] Lawn SD, Afful B, Acheampong JW (1998). Pulmonary tuberculosis: diagnostic delay in Ghanaian adults. Int J Tuberc Lung Dis.

[22] Chen HG (2014). Impact of diabetes on diagnostic delay for pulmonary tuberculosis in Beijing. Int J Tuberc Lung Dis.

[23] Sreeramareddy CT (2009). Time delays in diagnosis of pulmonary tuberculosis: a systematic review of literature. BMC Infect Dis.

[24] QGIS Development team (2015). QGIS Geographic Information System. Open Source Geospatial Foundation Project.

[25] Coveney J (2015). FIRTHLOGIT: Stata module to calculate bias reduction in logistic regression. Statistical Software Components.

[26] Yimer SA, Bjune GA, Holm-Hansen C (2014). Time to first consultation, diagnosis and treatment of TB among patients attending a referral hospital in Northwest, Ethiopia. BMC Infect Dis.

[27] Thakur R, Murhekar M (2013). Delay in diagnosis and treatment among TB patients registered under RNTCP Mandi, Himachal Pradesh, India, 2010. Indian J Tuberc.

[28] Saifodine A (2013). Patient and health system delay among patients with pulmonary tuberculosis in Beira city, Mozambique. BMC Public Health.

[29] Mfinanga SG (2008). The magnitude and factors associated with delays in management of smear positive tuberculosis in Dar es Salaam, Tanzania. BMC Health Serv Res.

[30] Irani L, Kabalimu TK, Kasesela S (2007). Knowledge and healthcare seeking behaviour of pulmonary tuberculosis patients attending Ilala District Hospital, Tanzania. Tanzan Health Res Bull.

[31] Hinderaker SG (2011). Treatment delay among tuberculosis patients in Tanzania: data from the FIDELIS initiative. BMC Public Health.

[32] Lambert ML, Van der Stuyft P (2005). Delays to tuberculosis treatment: shall we continue to blame the victim?. Trop Med Int Health.

[33] Shete PB (2015). Pathways and costs of care for patients with tuberculosis symptoms in rural Uganda. Int J Tuberc Lung Dis.

[34] Odusanya OO, Babafemi JO (2004). Patterns of delays amongst pulmonary tuberculosis patients in Lagos, Nigeria. BMC Public Health.

[35] Biya O (2014). Knowledge, care-seeking behavior, and factors associated with patient delay among newly-diagnosed pulmonary tuberculosis patients, Federal Capital Territory, Nigeria, 2010. Pan Afr Med J.

[36] Paz-Soldan VA (2014). Patient Reported Delays in Seeking Treatment for Tuberculosis among Adult and Pediatric TB Patients and TB Patients Co-Infected with HIV in Lima, Peru: A Qualitative Study. Front Public Health.

[37] Belay M (2012). Diagnostic and treatment delay among Tuberculosis patients in Afar Region, Ethiopia: a cross-sectional study. BMC Public Health.

[38] Segagni Lusignani L (2013). Factors associated with patient and health care system delay in diagnosis for tuberculosis in the province of Luanda, Angola. BMC Infect Dis.

[39] Makwakwa L (2014). Patient and heath system delays in the diagnosis and treatment of new and retreatment pulmonary tuberculosis cases in Malawi. BMC Infect Dis.

[40] Ukwaja KN (2013). Healthcare-seeking behavior, treatment delays and its determinants among pulmonary tuberculosis patients in rural Nigeria: a cross-sectional study. BMC Health Serv Res.

[41] Sabawoon W, Sato H, Kobayashi Y (2012). Delay in the treatment of pulmonary tuberculosis: a report from Afghanistan. Environ Health Prev Med.

[42] Maciel EL (2010). Delay in diagnosis of pulmonary tuberculosis at a primary health clinic in Vitoria, Brazil. Int J Tuberc Lung Dis.

[43] Kardjito T, Grange JM (1980). Immunological and clinical features of smear-positive pulmonary tuberculosis in East Java. Tubercle.

[44] Cai J (2015). Factors associated with patient and provider delays for tuberculosis diagnosis and treatment in Asia: a systematic review and meta-analysis. PLoS ONE.

[45] Sabde YD (2011). Mapping private pharmacies and their characteristics in Ujjain district, Central India. BMC Health Serv Res.

[46] Theron G (2015). Data for action: collection and use of local data to end tuberculosis. Lancet.

[47] MacPherson P (2013). Development and validation of a global positioning system-based “map book” system for categorizing cluster residency status of community members living in high-density urban slums in Blantyre, Malawi. Am J Epidemiol.

[48] Hargreaves JR (2011). The social determinants of tuberculosis: from evidence to action. Am J Public Health.

[49] Lonnroth K (2009). Drivers of tuberculosis epidemics: the role of risk factors and social determinants. Soc Sci Med.

[50] Maske AP (2015). Socio-cultural features and help-seeking preferences for leprosy and turbeculosis: a cultural epidemiological study in a tribal district of Maharashtra, India. Infect Dis Poverty.

