# Supplementary material for: Diagnostic delay and associated factors among patients with pulmonary tuberculosis in Dar es Salaam, Tanzania
Source: Infect Dis Poverty. 2017 Mar 24;6:64. doi: 10.1186/s40249-017-0276-4 (PMC5364704; doi:10.1186/s40249-017-0276-4)
Supplement: Supplementary file 1 — Multilingual abstracts in the five official working languages of the United Nations. (PDF 776 kb) [file 40249_2017_276_MOESM1_ESM.pdf]

Translation of the abstract into the five official working languages of the United Nations

تأخير التشخيص والعوامل المرتبطة به بين المرضى الذين يعانون من مرض السل الرئوي في دار السلام، تنزانيا

خديجة سعيد، جيري هيلا، جريس مهالو، ماري تشيريانكوبي، إدوارد ماسيكا، توماس مارو، فرانسيس مهيمبيرا، نعمه كبالاتا ولوكاس فنر

خلفية: تنزانيا من بين الدول الثلاثين الأعلى في إصابات السل (TB). ولأن للسل فترة عدوى طويلة، فالتشخيص المبكر مهم ليس فقط للحد من انتقال العدوى، ولكن أيضا من أجل تحسين نتائج العلاج. قمنا بتقييم تأخير التشخيص والعوامل المرتبطة بها بين مرضى السل المعدي.

الأساليب: قابلنا مرضى السل الرئوي البالغين إيجابيي اللطاخة حديثي الإصابة المسجلين في دراسة أتراب للسل مستمرة في دار السلام، تنزانيا، بين نوفمبر 2013 ويونيو 2015. وأجريت مقابلات مع مرضى السل لجمع المعلومات حول التركيبة السكانية الاجتماعية، والوضع الاجتماعي والاقتصادي، سلوك الحصول على الرعاية الصحية، والرموز الديموغرافية السكنية. صنفنا تأخر التشخيص إلى ≤ 3 أو> 3 أسابيع. استخدمنا نماذج الانحدار اللوجستي لتحديد عوامل الخطر لتأخر التشخيص، وكانت نسب احتمالات معروضة في شكلها الأصلي (OR) معدلة (aOR). قمنا أيضا بتقييم العلاقة بين المسافة الجغرافية (الزيادة التدريجية من 500 متر بين الأسرة وأقرب صيدلية) مع النتائج الثنائية.

النتائج: حللنا حالات 513 مريض متوسط عمرهم 34 عاما (مجموعة الشرائح الربعية 27 - 41)؛ كان 353 (69٪) من الرجال. وعموما، ذكر 444 (87٪) أنهم طلبوا الرعاية من مقدمي الرعاية الصحية قبل تشخيص مرض السل، منهم 211 (48٪) سعى للحصول على الرعاية الصحية > 2 مرات. ستة فقط (1٪) زار المعالجين التقليديين قبل تشخيص مرض السل. وارتبط تأخر التشخيص الإيجابي مع عدم وجود ألم في الصدر (aOR 7.97، 95٪ فترات الثقة (CI) 3.15-20.19؛ P <0.001)، وجود بصق دموي (aOR 25.37، 95٪ CI11.15-57.74؛ P <0.001) ارتبط سلبيا مع استخدام الدواء قبل تشخيص مرض السل (aOR 0.31، 95٪ CI0.14-0.71؛ P = 0.01). العمر، والجنس، والإصابة بفيروس نقص المناعة البشرية، ومستوى التعليم ودخل الأسرة، وزيارة مرافق الرعاية الصحية لا ترتبط بتأخر التشخيص. كان المرضى الذين يعيشون بعيدا عن الصيدليات أقل عرضة لزيارة المنشآت الصحية (الزيادة التدريجية في المسافة مقابل زيارة أي منشأة: OR 0.51، 95٪ CI0.28-0.96؛ P = 0.037).

الاستنتاجات: تأخر تشخيص السل شائع في دار السلام، وكان ومرجح بين المرضى الذين لم يسبق لهم استخدام الأدوية مع بصق دموي. قد يكون للمسافة الجغرافية مع مرافق الرعاية الصحية تأثير على سلوك طلب الرعاية الصحة. يمكن لزيادة الوعي المجتمعي بعلامات السل وأعراضه أن تخفض تأخر التشخيص وتحد من انتقال مرض السل.

Translated from English version into Arabic by Mahmoud Sami, through

TRANSLATORS WITHOUT BORDERS

坦桑尼亚达累斯萨拉姆的肺结核病病人诊断延误及相关因素

Khadija Said, Jerry Hella, Grace Mhalu, Mary Chiryankubi, Edward Masika, Thomas Maroa, Francis Mhimbira, Neema Kapalata, Lukas Fenner

引言：坦桑尼亚是全球 30 个结核病负担最高的国家之一。由于结核病的传染期长，早期诊断不仅有利于减少传播，还能够改善治疗预后。我们分析了传染性结核病病人的诊断延误和相关因素。

方法：我们调查了 2013 年 11 月-2015 年 6 月正在坦桑尼亚达累斯萨拉姆进行的结核病队列研究中招募的新诊断的涂阳成年肺结核病病人。通过调查结核病病人，收集了社会人口学、社会经济学、求医行为和居住地区编码。诊断延误划分为 2 类：不超过 3 周和超过 3 周。采用 logistic 回归模型分析诊断延误的危险因素，采用粗比值比（*OR*）和调整的比值比（a*OR*）表示。同时分析了地理距离（家庭与最近药店的距离，以 500 米 1 个增量单位）与诊断延误的相关性。
结果：我们分析了 513 例病人，平均年龄 34 岁（四分位数：37-41），其中 353 例(69%)是男性。444 例（87%）诊断为结核病之前曾有求医应为，其中 211 例（48%）超过 2 次。仅有 6 例（1%）寻求过传统治疗方法。诊断延误与无胸痛表现呈正相关（a*OR*：7.97，95%可信区间[*CI*]：3.15 – 20.19；$P < 0.001$）；与出现咳血呈正相关（a*OR*：25.37，95% *CI*：11.15-57.74；$P < 0.001$）；与确诊前用药史呈负相关（a*OR*：0.31，95% *CI*：0.14-0.71;；$P = 0.01$）。年龄、性别、HIV 状况、教育水平、家庭收入、前往卫生服务中心求医等与诊断延误无关。病人住在离药店较远的地方前往卫生服务中心求医的可能性较低（前往任何医疗点的距离每增加 1 个单位，*OR* 为 0.51，95% *CI*：0.28-0.96；$P = 0.037$）。
结论：在达累斯萨拉姆结核病诊断延误普遍，尤其是在那些有用药史和出现咳血的病人。与卫生服务中心的距离对于求医行为有影响。提高社区对结核病的体征和症状的认识将有利于减少诊断延误以及阻断结核病的传播。

Translated from English version into Chinese by Men-Bao Qian, through

CHINESE CENTER FOR DISEASE CONTROL AND PREVENTION
NATIONAL INSTITUTE OF PARASITIC DISEASES

Retard de diagnostic et facteurs associés parmi les patients souffrant de tuberculose pulmonaire à Dar es Salaam, en Tanzanie

Khadija Said, Jerry Hella, Grace Mhalu, Mary Chiryankubi, Edward Masika, Thomas Maroa, Francis Mhimbira, Neema Kapalata, Lukas Fenner

Contexte : La Tanzanie est l'un des 30 pays où le fardeau de la tuberculose est le plus lourd. Parce que la période infectieuse de la tuberculose est longue, il est important de la diagnostiquer tôt, non seulement pour réduire la transmission mais aussi pour améliorer le résultat du traitement. Nous avons évalué le retard de diagnostic et les facteurs associés parmi des patients tuberculeux infectieux.
Méthodes : Nous avons questionné des patients adultes trouvés récemment positifs pour la tuberculose pulmonaire à l'examen des frottis et inclus dans une étude de cohortes sur la tuberculose à Dar es Salaam, en Tanzanie, entre novembre 2013 et juin 2015. Les entretiens avec les patients tuberculeux avaient pour but de recueillir des informations sur leur statut sociodémographique et socioéconomique, leurs comportements de recherche de soins et leur géocodes résidentiels. Nous avons catégorisé le retard de diagnostic en ≤ 3 et > 3 semaines. Nous avons utilisé des modèles de régression logistique pour identifier les facteurs de risque de retard de diagnostic, présentés sous la forme des rapports de cotes bruts (*OR*) et ajustés (*OR*a). Nous avons également évalué l'association entre la distance géographique (entre le domicile et la pharmacie la plus proche, par incréments de 500 mètres), avec des critères d'évaluation binaires.

Résultats : Nous avons analysé 513 patients d'un âge médian de 34 ans (fourchette interquartiles de 27 à 71, dont 353 hommes (69 %). Dans l'ensemble, 444 d'entre eux (87 %) ont rapporté avoir demandé des soins à des professionnels des soins de santé avant le diagnostic de tuberculose, plus de 2 fois dans 211 cas (48 %). Seuls six des sujets (1 %) étaient allés voir un tradipraticien avant le diagnostic de tuberculose. Le retard du diagnostic était associé de façon positive à l'absence de douleur thoracique (*OR*a 7,97, intervalle de confiance (IC) à 95 % de 3,15 à 20,19 ; $P < 0,001$) et à la présence d'hémoptysie (*OR*a 25,37, IC à 95 % de 11,15 à 57,74 ; $P < 0,001$) et de façon négative à la prise de médicaments avant le diagnostic de tuberculoses (*OR*a 0,31, IC à 95 % de 0,14 à 0,71 ; $P = 0,01$). L'âge, le sexe, le statut d'infection par le VIH, le niveau d'éducation, les revenus du ménage et la fréquentation de centres de soins de santé (CSS) n'étaient pas associés à un retard de diagnostic. Les patients vivant loin d'une pharmacie étaient moins enclins à se rendre dans un CSS (augmentation incrémentielle de la distance contre visite à un établissement quelconque : *OR* 0,51, IC à 95 % de 0,28 à 0,96, $P = 0,037$).
Conclusions : Le retard de diagnostic de la tuberculose s'est avéré fréquent à Dar es Salaam, et plus encore parmi les patients qui n'avaient pas pris de médicaments avant le dépistage et qui présentaient une hémoptysie. La distance géographique par rapport aux centres de soin de santé peut avoir un impact sur les comportements de recherche de soins. La sensibilisation croissante des communautés aux signes et symptômes de la tuberculose pourrait contribuer à réduire les retards de diagnostic et à interrompre la transmission de la maladie.

Translated from English version into French by Suzanne Assenat, through

TRANSLATORS
WITHOUT BORDERS

Диагностической задержки и связанные с ними факторы, среди больных туберкулезом легких в Dar es Salaam, Танзания

Khadija Said, Jerry Hella, Grace Mhalu, Mary Chiryankubi, Edward Masika, Thomas Maroa, Francis Mhimbira, Neema Kapalata, Lukas Fenner

Фон: Танзания входит в число 30 стран с самым высоким числом туберкулеза (ТВ). Потому что туберкулез имеет долгий инфекционный период, ранняя диагностика имеет важное значение не только для снижения уровня передачи, а также для улучшения результатов лечения. Мы провели оценку диагностической задержки и связанные с ними факторы, среди заразных больных ТВ.
Методы: Мы взяли интервью у новых больных с положительным мазком легочной взрослый ТВ поступил в исследовании когорты ТВ в Dar es Salaam, Танзания в период с ноября 2013 по июнь 2015 года. Больные туберкулезом были проведены беседы для сбора информации о социально-демографических, социально-экономических статусом, здоровье, поведении и жилых геокодах. Мы выявили диагностической задержки в ≤ 3 или > 3 недель. Мы использовали модели логистической регрессии для выявления факторов риска поздней диагностики, представленные в виде сырой (*OR*) и скорректированного Odds Ratios (a*OR*).

Мы также оценили связь между географическим расстоянием (постепенное увеличение в 500 метров между бытовыми и ближайшей аптекой) с бинарными исходами.
Результаты: Мы проанализировали 513 пациентов со средним возрастом 34 года (межквартильный диапазон 27 – 41); 353 (69%) были мужчины. В целом, 444 (87%) сообщили поиск помощи от медицинских работников до диагностики туберкулеза, из которых 211 (48%) обращались за помощью > 2 раза. Только шесть (1%) посещали народных целителей до постановки диагноза ТВ. Диагностическая задержка была положительно связана с отсутствием боли в груди (a*OR* 7.97, 95% доверительный интервал [*CI*] 3.15 – 20.19; $P <$ 0,001), и наличие кровохарканья (a*OR* 25.37, 95% *CI* 11.15-57.74; $P <$ 0,001) и отрицательно связана с использованием лекарств до диагностики туберкулеза (a*OR* 0.31, 95% *CI* 0.14-0.71; $P =$ 0,01). Возраст, пол, статус ВИЧ, уровень образования, семейный доход, и посещение медицинских учреждений (HCFs) не были связаны с диагностической задержкой. Пациенты живут далеко от аптеки, стали реже посещать HCFs (постепенное увеличение дистанции против посещения любого объекта: или 0,51, 95% *CI* 0,28-0,96; $P =$ 0.037).
Заключеие: Задержка диагностики ТВ была распространена в Dar es Salaam, и, скорее всего, среди пациентов без использования лекарств и представления с кровохарканьем. Географическое расстояние от HCFs может оказывать влияние на здоровье поведение. Повышение осведомленности сообщества знаки ТВ и симптомы могут способствовать дальнейшему снижению диагностической задержки и прерыванию передачи ТВ.

Translated from English version into Russian by Hao-Qi Zhang.

Dilación en el diagnóstico y factores asociados para pacientes con tuberculosis pulmonar en Dar es Salaam, Tanzania

Khadija Said, Jerry Hella, Grace Mhalu, María Chiryankubi, Edward Masika, Thomas Maroa, Francisco Mhimbira, Neema Kapalata, Lukas Fenner

Referencias: Tanzania es uno de los 30 países con mayor censo en su población que padece de tuberculosis (TB). Debido a que la tuberculosis tiene un largo período infeccioso, no sólo es importante un diagnóstico precoz para reducir la transmisión, sino también para mejorar los resultados en el tratamiento. Se evaluó la dilación en el diagnóstico así como los factores asociados en pacientes con tuberculosis infecciosa.
Metodologías: Entre noviembre de 2013 y junio de 2015, se entrevistó a nuevos pacientes que padecían de tuberculosis pulmonar de frotis positivo, quienes estaban registrados en un estudio en serie - todavía en desarrollo - sobre tuberculosis en Dar es Salaam, Tanzania. Se entrevistó a pacientes tuberculosos para recopilar información socio-demográfica, socio-económica, la conducta a la hora de buscar atención sanitaria y geo-códigos residenciales. Se clasificó por categorías la dilación en el diagnóstico en ≤ 3 o> 3 semanas. Se utilizaron modelos de retracción logística para identificar aquellos factores de riesgo concernientes a la dilación del diagnóstico, los cuales se presentaron como: Simples (OR; *por sus siglas en inglés*) y Radios de Probabilidad equilibrada (aOR; *por sus siglas en inglés*). También pudimos evaluar una asociación entre la distancia geográfica (aumento incremental de 500 metros entre el hogar y la farmacia más cercana)

obteniendo resultados binarios.

Resultados: Se analizaron a 513 pacientes, con un promedio de edad de 34 años (rangos intercuartiles 27 - 41); 353 (69%) eran hombres. En general, 444 (87%) nos informaron que buscaron atención médica antes de ser diagnosticadas de tuberculosis, de los cuales 211 (48%) buscaron asistencia> 2 veces. Sólo seis (1%) visitaron a curanderos tradicionales, antes que se les diagnosticara la tuberculosis. La dilación del diagnóstico se asoció positivamente con la ausencia de dolor torácico (aOR 7,97, Intervalos de Confidencia del 95% [IC] 3,15 - 20,19, P <0,001) y presencia de hemoptisis (aOR 25,37, IC 95% 11,15-57,74; P <0,001) la cual se encontraba asociada negativamente con el uso de la medicación, antes del diagnóstico de la tuberculosis (ORa 0,31; IC del 95%: 0,14 a 0,71; P = 0,01). La edad, el sexo, la condición de VIH, el nivel de educación, los ingresos en el hogar y los centros sanitarios que visitaron y las Instalaciones de Asistencia Sanitaria (HCF; *por sus siglas en inglés*) no se asociaron con la dilación en el diagnóstico. Los pacientes que vivían lejos de las farmacias tuvieron menos probabilidades de visitar un HCF (aumento incremental de la distancia versus visita a cualquier centro sanitario: OR 0,51; IC del 95%: 0,28-0,96; P = 0,037).

Conclusiones: La dilación para el diagnóstico de la tuberculosis fue común en Dar es Salaam, y más probable entre los pacientes sin un uso previo de medicación presentando hemoptisis. La distancia geográfica a las HCF puede tener un impacto en el comportamiento, a la hora de buscar asistencia sanitaria. El aumento de la toma de conciencia social sobre las indicaciones y síntomas de la tuberculosis podría reducir, aún más, las dilaciones del diagnóstico e interrumpir la transmisión de la tuberculosis.

Translated from English version into Spanish by Maria Luz Puert, through

TRANSLATORS
WITHOUT BORDERS
