# Supplementary material for: Diagnostic delay and associated factors among patients with pulmonary tuberculosis in Dar es Salaam, Tanzania
Source: Infect Dis Poverty. 2017 Mar 24;6:64. doi: 10.1186/s40249-017-0276-4 (PMC5364704; doi:10.1186/s40249-017-0276-4)
Supplement: Supplementary file 2 — Histogram of delay time reported among 507 TB patients. (DOCX 79 kb) [file 40249_2017_276_MOESM2_ESM.docx]

**Additional file**

**Diagnostic delay and associated factors among patients with pulmonary tuberculosis in Dar es Salaam, Tanzania**

Authors: Khadija Said, Jerry Hella, Grace Mhalu, Mary Chiryankubi, Edward Masika, Thomas Maroa, Francis Mhimbira, Neema Kapalata, Lukas Fenner

**SUPPLEMENTARY MATERIALS**

**Additional file 1.**

**Figure S1:** Histogram of delay time reported among 507 TB patients.

Six patients with delays of >5 weeks were excluded in this figure (1 patient with a delay of 6 weeks, 1 patient with 8 weeks, and 3 patients with 12 weeks, and 1 patient with a delay of 45 weeks)
